# Chronic exposure to ∆ ^9^-tetrahydrocannabinol in adolescence decreases social play behaviours

**DOI:** 10.12688/f1000research.53891.1

**Published:** 2021-11-24

**Authors:** Robin Keeley, Stephanie Himmler, Sergio Pellis, Robert McDonald

**Affiliations:** 1 Canadian Centre for Behavioural Neuroscience, The University of Lethbridge, Lethbridge, AB, T1K 3M4, Canada

**Keywords:** THC, adolescence, rat, play, social behaviour

## Abstract

**Background**: Cannabis use remains a major public health concern, and its use typically begins in adolescence. Chronic administration of ∆ ^9^-tetrahydrocannabinol (THC), the main psychoactive compound in cannabis, during adolescence can produce deficits in adult learning and memory, stress reactivity and anxiety. One possible mechanism behind the disruptions in adulthood from adolescent exposure to THC includes changes in social behaviours, such as social play, which has been shown to be critical to socio-cognitive development.

**Methods:** Here, using an established animal model of adolescent THC exposure in male and female Long–Evans rats, we explored the effects of THC on play behaviour during the chronic administration period. Following puberty onset, as indicated by external changes to the genitalia, THC (5mg/kg) was administered for 14 days. Play behaviour was assessed seven days following the onset of the injection period at approximately 1 hour post treatment. The frequency of nape attacks, the likelihood and tactics of defensive behaviour, and pins were scored and analyzed.

**Results:** THC exposure decreased playfulness in adolescent rats including the number of attacks, likelihood of defense and pins compared to control and vehicle treated rats. **Conclusion**: This suggests that THC suppresses both the attack and defense components of social play. This is an important finding because there is evidence that attack and defense may be mediated by different mechanisms. Furthermore, the effect of THC exposure decreasing playfulness occurred similarly in males and females.

## Introduction

The first instance of cannabis use typically begins in adolescence, ^1^ although the long-term consequences of adolescent cannabis exposure are not clear. Δ ^9^-tetrahydrocannabinol (THC) is the main psychoactive component in cannabis, ^2^ and accounts for many of its psychoactive properties. ^3^ Chronic THC administration in adolescent rats can produce genetic background- and sex-specific deficits in adult learning and memory, stress reactivity and anxiety, ^4^ although the mechanism producing the behavioural changes observed in adults exposed to THC as adolescents is poorly understood.

Adolescence is an evolutionarily conserved period of brain development, ^5^ and pharmacological or behavioural perturbations of this critical developmental period can induce long-term changes in adult brain and behaviour, specific to the background and sex of the individual. ^6^ However, the complex interplay between adolescent manipulations and resultant changes in social behaviours remains underexplored [for review, see Ref. 7]. Social play peaks during the juvenile period ^8^ and is critical to the development of socio-cognitive skills and their underlying brain mechanisms. ^9^

Acute administration of cannabinoids decreases social play, ^10^ and chronic treatment with cannabinoids in adolescence can decrease play behaviour in adulthood. ^11^ Given that our earlier research identified some long-term changes to adult brain and behaviour following adolescent THC exposure, it is crucial to understand whether these changes were purely pharmacological or involved a complex interplay between pharmacology and behaviour. Furthermore, the way in which play, which involves competitive wrestling, ^12^ has been measured by most studies [for example, Ref. 10] in ways that do not allow the detailed assessment of which aspects of play change with treatment. Where such measures have been expanded to include direct assessment of attack and defense, there is some indication that some aspects of defense may be especially susceptible. ^11^ The present study also seeks to expand the current literature by identifying whether all aspects of play or only specific aspects of play are affected by chronic exposure to THC. Here, we used a previously established animal model of adolescent drug exposure ^4^^,^^13^ in male and female Long–Evans rats to explore the effects of THC on play behaviour during the chronic administration period.

## Methods

A total of 48 Long–Evans rats (24 females and 24 males; RRID: RGD_18337282) were bred in-house at the Canadian Centre for Behavioural Neuroscience (CCBN) at the University of Lethbridge. Sample size was determined using a combination of prior literature ^14^ as well as a power analysis. No animals were excluded from this analysis. All rats were placed in standard home cages (46×25×20 cm; maintained at 21°C and 35% relative humidity) and were given *ad libitum* access to food and water. Rats were weaned at postnatal day 21 and pseudo-randomly placed in same-sex quadrads, with no more than two littermates per quadrad. Following weaning, male and female rats were randomly assigned and counterbalanced to their experimental groups: comparison control (CC; N = 6), control (CON; N = 6) vehicle (VEH; N = 6) or THC (N = 6) so that each quadrad included one same-sex partner subject from each experimental group. Blinding occurred during group allocation as the person assigning the rats to each quadrad did not know the eventual treatment group of each rat. The CC group received handling like the CON group, but they were the play partner with which all other group members interacted. For each play bout measured, the behaviour of CON, VEH and THC treated rats was quantified. All procedures were done in accordance with the Canadian Council on Animal Care and were approved by the University of Lethbridge Animal Welfare Committee. Additionally, all experimental procedures were carried out according to the Institutional Animal Care and Usage Committee (ARRIVE guidelines).

Treatment began following puberty onset as previously described, ^13^ using the external changes to the genitalia, which reliably signal the onset of puberty. ^4^ THC (in 1:1:18 solution of ethanol: cremaphor:saline) was administered *i.p.* at a dose of 5 mg/kg once a day starting on the day of puberty onset for 14 days. This dose of THC was chosen given that it can produce both acute and chronic effects on brain and behavior. VEH groups were given a vehicle injection. All injections were performed during the last third of the dark cycle. On each injection day, rats were removed from their home-cages and placed in a light-blocking transport tub and brought to a procedural room that was lit with a red incandescent bulb. All rats were weighed before treatment. For VEH and THC rats, the injection site was varied daily to eliminate any damage and/or irritation due to multiple injections at the same site. Following injections, all rats were brought back to their home-cages. All treatment groups were handled for approximately 5 min.

Play behaviour was tested using previously described procedures. ^14^ Testing consisted of placing two play partners in large, clear Plexiglas arena (5050 × 50 cm), filled to a depth of ~1–2 cm with Betacob bedding. Play was recorded for 10 min in the dark using a video camera with night-shot capacity. Following the trial, rats were returned to their home cage. No adverse events were reported. Upon completion of the experiment, the rats were humanely sacrificed with an *i.p.* injection of sodium pentobarbital (300 mg/kg).

**Figure 1.**
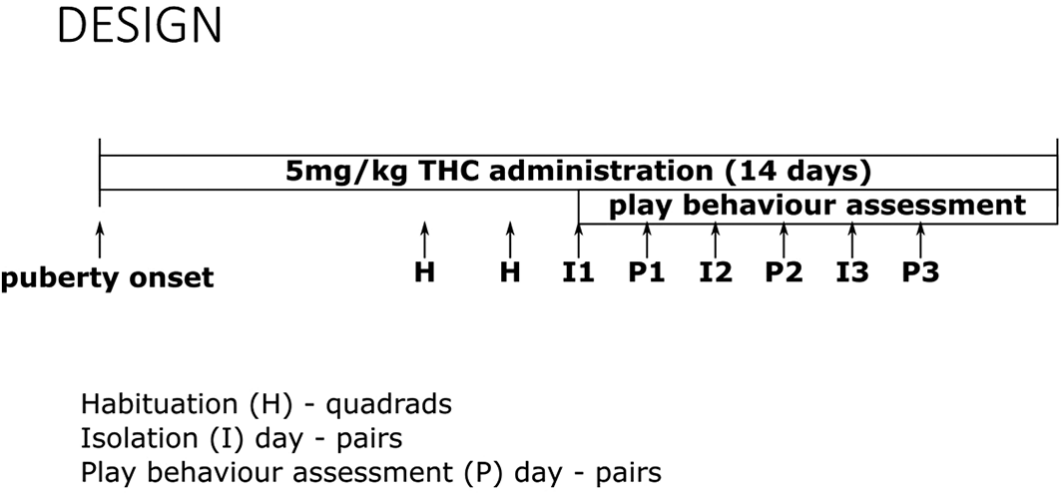
Experimental timeline. Rats began THC exposure at puberty onset. On days 5 and 6, rat quadrads were habituated (H) to the play apparatus. On day 7, pairs of rats were isolated (I), always including the comparison control group, to which all other rats were paired. Play behaviour (P) was assessed on subsequent days. For all quadrads, there were three isolation days and three play behavior assessments to assess all pairings of treatment groups with the comparison control (CC–CON, CC–VEH and CC–THC).

A timeline of drug treatment and play behaviour assessment can be found in Figure 1. Play behaviour was assessed at least seven days following the onset of the injection period ~1 hour after treatment. Since we were interested in the acute effects of THC behaviour during the chronic administration period, we chose a time-point wherein the rats would be habituated to the treatment while still acutely intoxicated. On day 5 and 6 of treatment, quadrads were habituated to the play apparatus for 30 min. 24 hours before play trials, partners were separated and housed individually. To reduce isolation distress during this time period, all of the rats had access to a black tube and shredded paper for enrichment. Within a quadrad, all treatment rats (CON, VEH and THC) were paired with the CC rat for play bouts. The order of play bouts with the CC was counterbalanced, the play apparatus was cleaned between recordings using Virkon, and fresh bedding was replaced for each session.

Videos were analyzed using previously described techniques. ^14^ The experimenter scoring the videos were blind to the experimental groups, however, the THC group’s behaviour may have been different compared to the other groups and therefore complete concealment may not have been obtained. During play, rats compete to contact and nuzzle the partner’s nape of the neck. ^12^ The frequency of nape attacks (in which the snout of one partner moves towards the nape of the other), the likelihood of the partner receiving a nape attack defending itself to avoid contact and if it did defend itself, the tactics used to do so were scored. ^14^ The total frequency of nape attacks per 10 min were scored and analyzed, but as defense is contingent on attack, the frequency of defense was expressed as a percentage of attacks received, and the types of defense were expressed as a percentage of defended attacks. Defense can involve either evasion (the head and neck are turned away from the attacker) or facing defense (the defender turns to face the attacker). The percentage of evasion provides a measure of attempts by rats to avoid playful contact. In facing defense, the rats can either remain standing or roll over to supine, with the percentage of the latter providing a measure of the rat’s motivation to gain and maintain close body contact. A common configuration resulting from attack and defense in the juvenile period is for one rat to lie on its back and the partner to stand over it ( *i.e.*, a pin). ^15^ As the absolute number of pins per trial can provide insight into the pattern of interaction, ^16^ this was also scored. Data from this study has been archived. ^32^

All statistical analyses used R (version 3.4.1; RRID:SCR_001905). Main effects of group, sex and group by sex interactions were examined for their effect on the frequency of nape attacks and pins as well as the percentage of defensive behaviours, the percentage of evasions (as a percentage of defended attacks) and the percentage of complete rotations (as a percentage of facing defenses). *Post hoc* comparisons were conducted when main effects or interactions were observed using Bonferonni corrections.

## Results

**Figure 2.**
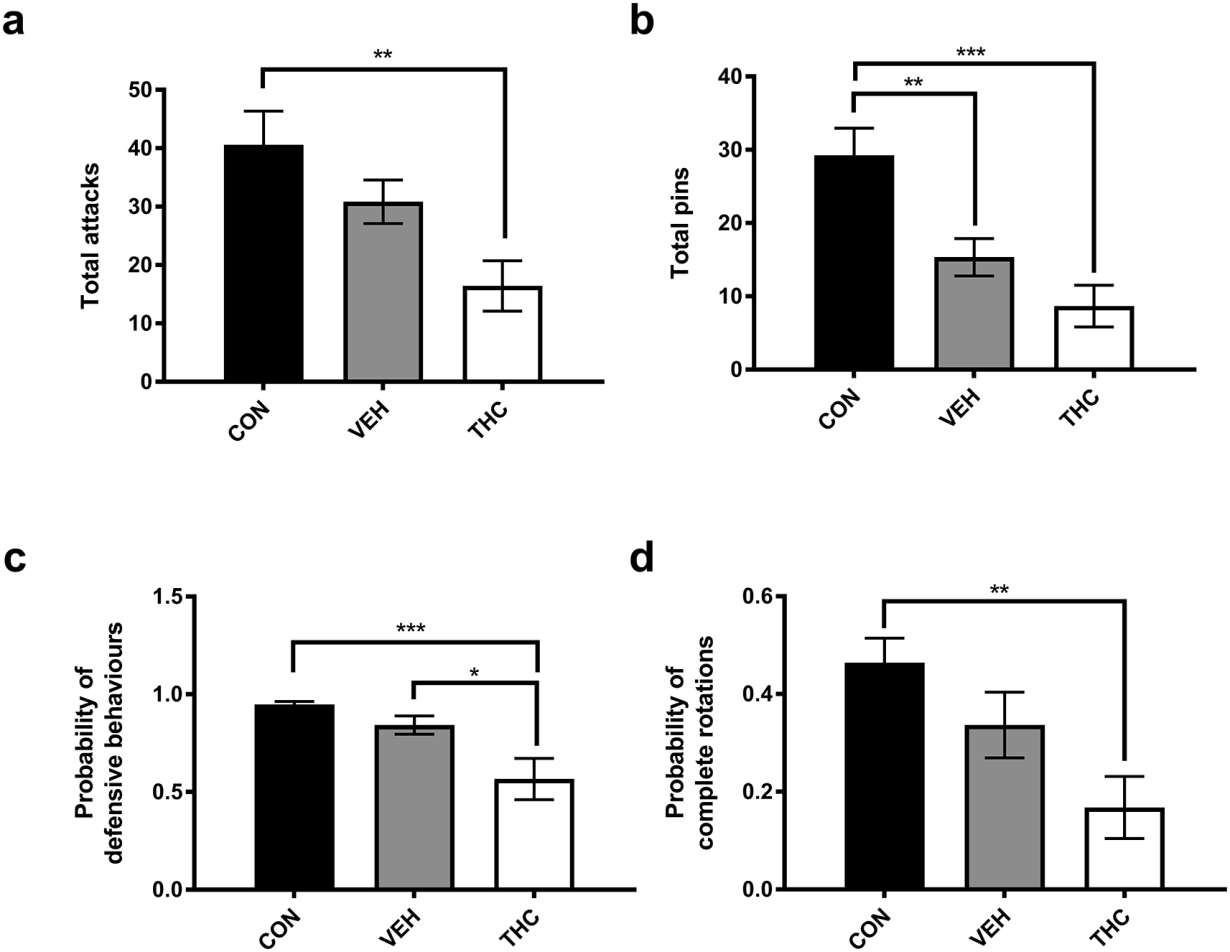
a) Total number of attacks. b) Total number of pins. c) Probability of defensive behaviours. d) Probability of complete rotations. * *p* < 0.05. ** *p* < 0.01. ** *p* < 0.001.

For all analyses, there was no significant effect of sex or an interaction between treatment group and sex ( *p*’s > 0.05). Mean and SEM for all treatment groups as well as statistical output for the main effect of treatment and *post hoc* comparisons can be found in Table 1. There was a significant effect of treatment group on the total number of nape attacks (F _(2,30)_ = 6.55, *p* = 0.00434; Figure 2a) and pins (F _(2,30)_ = 11.61, *p* = 0.000184; Figure 2b). *Post hoc* comparisons between groups revealed that CON rats attacked ( *p* = 0.0027) and pinned ( *p* = 0.00012) significantly more than THC-exposed rats. Further, CON rats pinned significantly more than VEH-exposed rats ( *p* = 0.0090). There was a significant effect of treatment group on the likelihood of defense (F _(2,30)_ = 8.24, *p* = 0.0014). CON ( *p* = 0.00096) and VEH ( *p* = 0.019) rats were significantly more likely to defend themselves than the THC-treatment group ( Figure 2c). There was a significant group effect on the likelihood of complete rotations (F _(2,30)_ = 6.08, *p* = 0.00608), with CON rats engaging in significantly more complete rotations as compared to THC-treated ( *p* = 0.0046) rats ( Figure 2d). There was no significant group effect on the likelihood of evasion ( Table 1).

**Table 1.** Mean and standard errors for all treatment groups, including statistical test of the main effect of treatment.

Behaviour	Mean±SEM			Main effect of treatment				
	CON	VEH	THC	F	p	*Post hoc* Bonferroni		
						CON *vs* VEH	CON *vs* THC	VEH *vs* THC
Total attacks (Frequency)	40.6 ± 5.77	30.8 ± 3.71	16.4 ± 4.32	6.55	0.0044	n.s.	**	n.s.
Total pins (Frequency)	29.2 ± 3.70	15.3 ± 2.55	8.67 ± 2.85	11.61	0.00018	**	***	n.s.
% Defense	0.948 ± 0.0148	0.843 ± 0.0468	0.567 ± 0.105	8.24	0.0014	n.s.	***	*
% Evasion	0.375 ± 0.0494	0.482 ± 0.0543	0.519 ± 0.106	0.94	0.40	n.s.	n.s.	n.s.
% Complete rotation	0.464 ± 0.0500	0.337 ± 0.0672	0.168 ± 0.0634	6.08	0.0061	n.s.	**	n.s.

Note: n.s. denotes not significant.

* *p* < 0.05.

** *p* < 0.01.

*** *p* < 0.001.

Here, we assessed the effects of adolescent exposure to THC on play behaviours. Adolescent exposure to THC decreased attacks, pins and overall defensive behaviour. There was a significant reduction in the VEH-treated groups in the total number of pins. There are two possible explanations for this effect. First, injection stress could decrease overall playfulness. This is unlikely as injections of other play suppressing drugs have had significantly greater effects than injections of saline. ^17^ Second, our vehicle contained alcohol, which, when administered chronically, suppresses adolescent play. ^18^ However, when administered acutely at low doses, alcohol can facilitate play. ^19^ Therefore, it is unclear whether the small doses of alcohol used in the vehicle for the present study would by itself suppress play. Still, although the possibility of a synergistic effect by combining THC and alcohol cannot be discounted, it seems highly unlikely. Nonetheless, where both the VEH and THC had an effect, the effects of THC were greater ( Table 1, Figure 2), suggesting that the drug had an effect beyond that produced by the stress of injection or the added effects of low levels alcohol in the vehicle.

## Discussion/conclusions

Relative to the control condition, both the THC and the VEH groups had a lower likelihood that they would defend themselves when attacked. Combined with the reduced likelihood of initiating attacks by the THC-treated animals, this suggests that THC suppresses both the attack and defense components of social play. This is an important finding because there is evidence that attack and defense may be mediated by different mechanisms. ^16^ Also, the effect on how the THC rats defend themselves relative to controls is not uniform. The THC rats are just as likely to evade nape contact, but if they turn to face the attacker they are less likely to roll over to supine. The reduced likelihood of rolling to supine in combination with an overall reduced likelihood of defending themselves can account for the reduced frequency of pinning, as in this strain of rats the majority of pins arise from the defender turning to supine. ^20^ These changes are important in assessing the effects of THC because one possible mechanism is to make the animals lethargic, and so less inclined to attack, defend or facilitate the continued wrestling that is often accompanied by rolling over to the supine position. ^14^ However, THC did not change the likelihood to evade attacks, a maneuver that can involve rapid swerving, jumping and running, ^12^ so it is unlikely that THC induced lethargy. This is further supported by findings that THC-induced hypolocomotion is typically only observed after acute, ^21^ but not repeated and chronic exposure to THC. ^22^^–^^24^ Moreover, as complete rotation is calculated as a percentage of facing defense, a reduction in the percentage of complete rotation signals a concomitant increase in standing defense, ^14^ which require a significant amount of energy expenditure as the animals’ push, grapple and kick one another. ^12^ Additionally, any changes in play behaviour are not likely due to this dose of THC causing aversion, as 5 mg/kg of THC does not consistently produce aversion in standard place preference tasks. ^25^ Thus, while the endocannabinoid system constitutes an important part of the play–reward system, ^26^ it appears to have a selective effect on different aspects of what animals find rewarding during play.

Disrupting sociality in adolescence alters multiple neurobiobehavioural metrics associated with anxiety, depression and substance abuse, ^7^ and reduced exposure to play, such as through housing with an older conspecific, disrupts development of emotional regulation, cognition and sociality [as reviewed in Ref. 27]. The effects of a pharmacological manipulation that changes play behaviour could, in theory, result in secondary effects on brain development and adult behaviour, outside of the primary mechanism of the pharmacological manipulation. Supporting this possibility are studies with the use of different drugs in adolescence showing long lasting effects on brain and behaviour outside of their effects on play. ^28^^,^^29^

Previous studies have found differences in play behaviour as a function of acute cannabinoid manipulation [for example, Ref. 10]. Social play itself induces endocannabinoid release in the amygdala and nucleus accumbens, ^30^ thought to be washed out with systemic administration of THC, and the psychoactive properties of THC may block performance of complex behaviours like play. ^31^ Thus, THC administration acutely decreases playfulness; although to date, ours is the first study to demonstrate that this decrease in playfulness persists through a chronic administration period, which could mediate the effects of THC on adult brain volume and behaviour observed using this same administration paradigm. ^4^ These results suggest that effects of THC on subsequent adult brain and behaviour could be partially explained by an interaction between changing playfulness and the pharmacological effects of THC.

Although these results have important implications for understanding the effects of adolescent cannabis use, limitations remain. We measured in detail one aspect of sociality, social play. Inclusion of additional measures of social behaviours could have indicated global deficits or compensatory engagement in other social behaviours. Furthermore, our measures did not examine the temporal profile of the observed decreases in play, leaving open the possibility that THC-exposed adolescent rats could increase their playfulness once they are not acutely under the drug’s influence. Repeated assessment of play behaviour throughout the drug-administration period would have elucidated whether the observed effects were truly due to chronic exposure to THC or were merely affecting behaviours as a series of acute manipulations with no carryover from day-to-day. In addition, we did not measure any behavioural changes in adults that had been exposed to THC in adolescence, potentially demonstrating correlational relationships among play behaviour and sociality in adulthood. It remains unclear whether these changes in rat play behaviour during adolescence mediate causal influence on adult brain and behaviour. Finally, although we did not detect a sex difference of the effects of THC on play behaviour, it is possible that our study was underpowered to detect sex differences.

Here, we observed marked decreases in playfulness following acute administration of THC during a chronic administration period. We believe that this decrease in sociality could partially mediate some of the effects of THC in adolescence on adult brain and behaviour function. This study is especially important given the large number of adolescent humans that consume cannabis recreationally. Disruptions in social behaviour during this period could have long-term ramifications on adult brain and behaviour, and future research should consider the intersection between these two factors.

## Data availability

### Underlying data

Dryad: Underlying data for ‘Chronic exposure to ∆ ^9^-tetrahydrocannabinol in adolescence decreases social play behaviours’. https://doi.org/10.5061/dryad.v9s4mw6x4. ^32^

The project contains the following underlying data:

THC_and_play_data_set_2021.csv

This_DATSETNAMEreadme_THC_and_Play_Oct_29.docx

## Reporting guidelines

Dryad: ARRIVE checklist for ‘Chronic exposure to ∆9-tetrahydrocannabinol in adolescence decreases social play behaviours’.

DOI: https://doi.org/10.5061/dryad.v9s4mw6x4

Data are available under the terms of the Creative Commons Zero “No rights reserved” data waiver (CC0 1.0 Public domain dedication).

## Acknowledgements

We thank Brett Himmler and Vivien Pellis for comments and suggestions on earlier drafts of the paper.
