# Supplementary material for: Chronic exposure to ∆ 9-tetrahydrocannabinol in adolescence decreases social play behaviours
Source: F1000Res. 2021 Nov 24;10:1191. [Version 1] doi: 10.12688/f1000research.53891.1 (PMC8689401; doi:10.12688/f1000research.53891.1)
Supplement: Peer review history

# Peer review: Reviewer response for version 1

**DOI:** 10.5256/f1000research.57318.r101087

**Published:** 2022-01-07

**Authors:** Robin Murphy

**Affiliations:** 1 Department of Experimental Psychology, University of Oxford, Oxford, UK

This is an interesting piece of research, carefully conducted, analysed and reported. I have a few comments:

1. I thought some of the introductory and concluding comments emphasising the public health concern was probably not necessary, but perhaps more importantly the attempt to classify behavioural effects as disruptions or impairments was perhaps premature. This paper seems to take a stronger negative perspective than one of the papers cited to support the negative claims. Citation 4 reports both increases and decreases in performance on learning and memory tasks. It would perhaps be wise to take a slightly less decisive stance on this?
2. I have a similar comment about the concept of Play. Obviously play sounds good, but less good then attack and defence. The intro refers to play and the methods describe attacks. So playing devils advocate the the article could explain how THC supports lower aggression. I don't think I really mind which way, however, a more neutral stance might be preferred.
3. While it is true that attack and defence involve different neural pathways, and therefore if there is an effect in both then that might suggest a separate mechanism, given that they are output via the same behaviour, the impact of THC might be at the same motor level, I'm not saying it does but I think you need to moderate that claim.
4. I think the point about % attacks and defence being related to the frequency of the opponents engagement does require some mention of the analysis of any differences in frequencies. Normally the reader should be reassured that there were no significant differences in the frequencies or if not what that direction was. I'm sorry if this is reported and I just missed it.
5. The conclusions highlight that strain and sex differences matter, it would be good to highlight this when explaining your choice of LER over say WR especially given the differences that the two strains may have with respect to learning and memory.
6. Finally and least importantly, the number of significant figures in the table was an annoyance. Choose 2 or 3 significant figures or a decimal number but at the moment the numbers seem formatted oddly.

Overall I think this is interesting research, I like the social play measure, and while I understand the narrative that reduced play leads to future impairments in learning and memory, I could also understand a narrative that suggested that THC resulted in more peaceful play, and call this disruption to normal behaviour rather than an impairment?

Is the work clearly and accurately presented and does it cite the current literature?

Yes

If applicable, is the statistical analysis and its interpretation appropriate?

Yes

Are all the source data underlying the results available to ensure full reproducibility?

Yes

Is the study design appropriate and is the work technically sound?

Yes

Are the conclusions drawn adequately supported by the results?

Yes

Are sufficient details of methods and analysis provided to allow replication by others?

Yes

Reviewer Expertise:

Behavioural Neuroscience

I confirm that I have read this submission and believe that I have an appropriate level of expertise to confirm that it is of an acceptable scientific standard.

# Peer review: Reviewer response for version 1

**DOI:** 10.5256/f1000research.57318.r101485

**Published:** 2021-12-20

**Authors:** Francesco Leri

**Affiliations:** 1 Department of Psychology and Collaborative Neuroscience, University of Guelph, Guelph, ON, Canada

The authors administered 5 mg/kg THC to adolescent males and females LE rats for 14 days. Aspects of play behaviour were assessed 1 hour following the injection, after 7 days from the beginning of the administration period.

 It is reported that THC decreased number of nape attacks, pins, as well as probability of defensive behaviours and complete rotations.

 This reviewer can offer a few minor comments to possibly improve the clarity of the manuscript.

1. The Introduction section could be expanded to clarify why play behaviour was selected for investigation and what evidence in people would lead to the current investigation in animals. It will be important to focus on adolescent behaviour because animals were not tested in adulthood.
2. The introduction indicates that it is crucial to understand whether changes in adult behaviour are purely pharmacological or involve a complex interplay between pharmacology and behaviour, but it is not clear how the current experiment addressed this question.
3. The introduction mentions background- and sex-specific deficits in adults, but does not elaborate on why Long-Evans were selected and does not expand of sex-differences. These two relevant issues are also not examined in the discussion section.
4. The introduction could describe play behaviours in rats.
5. The methods section does not seem to explain complete rotations.  This could be revised not only because this behaviour is reported in the results, but also because it is discussed as a measure of general motor activity.
6. The authors could report the ANOVA models employed to analyze the data, and could specify that data from males and females were pooled for subsequent analysis.
7. In the version of the manuscript that I reviewed, there is a paragraph in the results section beginning with "Here" that seems much more fitting to the discussion section.
8. The authors do a good job in addressing the issue of possible THC-induced hypo-activity. This said, additional data collected in locomotion chambers, in rats singly tested, may be needed to fully rule out this possible confound.
9. The authors also do a good job in addressing some of the limitations of the study. This said, they may need to indicate that their current findings are limited to a single THC dose, and to one dosing regimen.
10. Finally, as mentioned above, it may be value to discuss the lack of sex differences.

Is the work clearly and accurately presented and does it cite the current literature?

Partly

If applicable, is the statistical analysis and its interpretation appropriate?

Yes

Are all the source data underlying the results available to ensure full reproducibility?

Yes

Is the study design appropriate and is the work technically sound?

Yes

Are the conclusions drawn adequately supported by the results?

Yes

Are sufficient details of methods and analysis provided to allow replication by others?

Yes

Reviewer Expertise:

Behavioural Neuroscience, Neuropsychopharmacology.

I confirm that I have read this submission and believe that I have an appropriate level of expertise to confirm that it is of an acceptable scientific standard.

# Peer review: Reviewer response for version 1

**DOI:** 10.5256/f1000research.57318.r101085

**Published:** 2021-12-06

**Authors:** Ming Li

**Affiliations:** 1 University of Nebraska-Lincoln, Lincoln, NE, USA

This manuscript reported a study that investigated the effects of chronic THC (the main psychoactive ingredient in cannabis) exposure during the early adolescent period on social behavior in adolescent male and female rats. The authors used a previously established animal model of adolescent THC exposure in male and female Long–Evans rats and provided a detailed analysis of social play behaviors affected by THC treatment (5 mg/kg, ip). They found that THC exposure decreased several components of social behavior in adolescent rats including the number of attacks, and the likelihood of defense and pins compared to control and vehicle treated rats. This work is significant as it may reveal one possible mechanism by which adolescent THC exposure alters the brain development and function seen in adulthood, that is, by changing social behavior of affected animals and causing secondary effects on brain development and adult behavior.

 The study was straightforward and the methods and results were clearly presented. The rationale and treatment regimen were justified. One main strength of this study, and an often overlooked issue was its detailed analysis of social behaviors. With their analysis, they were able to determine the impaired social behavior by THC was not due to drug-induced lethargic reaction, and both the attack and defense components of social play were suppressed by THC. This latter finding is important as there is evidence that attack and defense may be mediated by different mechanisms. I also appreciate the authors’ comments on the limitations of this study. Overall it is a concise study that provides an important insight with regards to the neurodevelopmental impacts of adolescent THC exposure on adult brain and behavioral functions.

 I only have a few minor comments.

1. The authors mentioned that “The order of play bouts with the CC was counterbalanced”. Does this mean that on each test day, 2 from each group were tested?
2. How was “the likelihood of the partner receiving a nape attack defending itself to avoid contact” calculated? Was it the same as the “probability of defensive behaviors”?
3. Could the authors define more clearly what the defending “tactics” are?

Is the work clearly and accurately presented and does it cite the current literature?

Yes

If applicable, is the statistical analysis and its interpretation appropriate?

Yes

Are all the source data underlying the results available to ensure full reproducibility?

Yes

Is the study design appropriate and is the work technically sound?

Yes

Are the conclusions drawn adequately supported by the results?

Yes

Are sufficient details of methods and analysis provided to allow replication by others?

Partly

Reviewer Expertise:

Behavioral pharmacology

I confirm that I have read this submission and believe that I have an appropriate level of expertise to confirm that it is of an acceptable scientific standard.
